# A Method for Improving the Accuracy and Efficiency of Bacteriophage Genome Annotation

**DOI:** 10.3390/ijms20143391

**Published:** 2019-07-10

**Authors:** Alicia Salisbury, Philippos K. Tsourkas

**Affiliations:** School of Life Sciences, University of Nevada Las Vegas, Las Vegas, NV 89154, USA

**Keywords:** bacteriophages, genomics, genome annotation, SEA-PHAGES, Lambda

## Abstract

Bacteriophages are the most numerous entities on Earth. The number of sequenced phage genomes is approximately 8000 and increasing rapidly. Sequencing of a genome is followed by annotation, where genes, start codons, and functions are putatively identified. The mainstays of phage genome annotation are auto-annotation programs such as Glimmer and GeneMark. Due to the relatively small size of phage genomes, many groups choose to manually curate auto-annotation results to increase accuracy. An additional benefit of manual curation of auto-annotated phage genomes is that the process is amenable to be performed by students, and has been shown to improve student recruitment to the sciences. However, despite its greater accuracy and pedagogical value, manual curation suffers from high labor cost, lack of standardization and a degree of subjectivity in decision making, and susceptibility to mistakes. Here, we present a method developed in our lab that is designed to produce accurate annotations while reducing subjectivity and providing a degree of standardization in decision-making. We show that our method produces genome annotations more accurate than auto-annotation programs while retaining the pedagogical benefits of manual genome curation.

## 1. Introduction

Bacteriophages, or phages for short, are viruses that infect bacteria. Phages are the most numerous biological entities on Earth, estimated at 10^31^ particles in the biosphere in a 10:1 ratio with bacteria [1,2,3]. Phages play a key role in environmental regulation [4], microbiome regulation [5], and in microbial genetics by serving as vectors for horizontal gene transfer [4]. Historically, phages have featured in several seminal discoveries in molecular biology, and are the source of a large number of enzymes used in biology and biotechnology applications [4,6]. Eclipsed as treatment agents by antibiotics in the mid-20th century, interest in phages as treatment agents is growing rapidly as a result of the rise in antibiotic-resistant bacteria [7].

**Figure 1:**
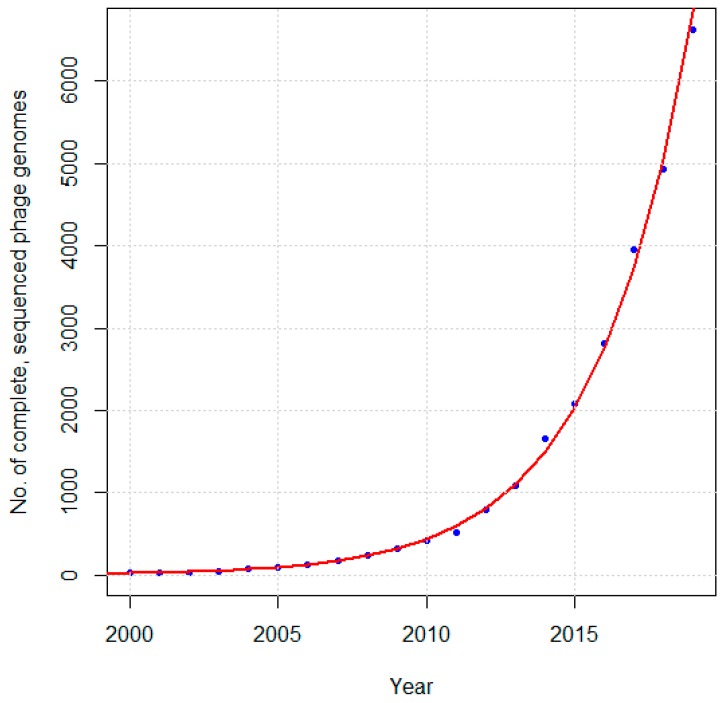
Cumulative number of unique, complete phage genome sequences in NCBI GenBank as of January 1 of each year since 2000. The red curve represents an exponential curve fit to the data (R^2^ = 0.989, *p*-value = 0).

Phage ΦX174 was the first organism to have its genome sequenced in its entirety, by Frederick Sanger and collaborators in 1977 [1,8]. Since then, the number of complete, sequenced phage genomes has grown rapidly, from 20 in 2000 [6], to 500 by 2008 [1], 750 by 2011 [2], 1000 by 2012 [9], 2000 by 2015 [10], to just over 8000 today (as determined by a search of NCBI Nucleotide with “Phage” and “Complete genome” in the “Title” search field, “Viruses” in the “Organism” filter, and “INSDC (GenBank)” in the “Source database” filter so as to not include duplicates due to RefSeq). As can be seen in Figure 1, the number of sequenced phage genomes is growing at an approximately exponential rate.

A significant number of sequenced phage genomes, approximately 2500 [11], are those of phages that infect *Actinobacteria*, isolated and sequenced with the support of the SEA-PHAGES program spearheaded by the University of Pittsburgh and Howard Hughes Medical Institute (HHMI) [12,13,14]. SEA-PHAGES provides support for an undergraduate research course offered at over 100 participating colleges and universities mainly in the USA, but also globally [12,13,14]. The course typically consists of two semesters: In the first semester, students isolate and characterize bacteriophages from environmental samples. Phage genomes are then sent to the University of Pittsburgh for sequencing and assembly. In the second semester, students annotate the assembled genomes and submit them to NCBI GenBank for archiving. The course has greatly increased the scientific community’s knowledge of actinobacteriophage diversity, while providing valuable scientific experience to thousands of students. Students that complete the course show higher interest and persistence in science, especially if they belong to groups traditionally underrepresented in the sciences [12,13,14].

The sequencing of an organism’s genome is followed by genome annotation, which consists of (1) the identification of genes, (2) the identification of the start codon of each gene, and (3) identification of putative gene function. For the vast majority of organisms, whether eukaryotic, prokaryotic, or viruses, genome annotation is performed in an automated fashion using one or more of several publicly available auto-annotation programs. For phages, a survey of the recent literature shows the most commonly used ab initio auto-annotation programs are Glimmer [15], the GeneMark family of programs [16,17,18,19,20] and Prodigal [21]. Other annotation packages, such as RAST [22] and Prokka [23] use one of these as their ab initio gene finder; RAST uses Glimmer while Prokka uses Prodigal. Recently, the program PHANOTATE was specifically designed to annotate phage genomes [24]. Although designed for bacterial genomes rather than phage genomes (with the exception of PHANOTATE), auto-annotation programs produce a complete and reasonably accurate annotation very rapidly. The downside is that auto-annotation programs occasionally produce false positives (non-coding open reading frames (ORFs) incorrectly identified as coding) and more frequently false negatives (undetected genes), and occasionally assign incorrect start codons [25]. This is problematic considering that the annotation of new phage genomes is often based on older annotations of similar phages, which results in the perpetuation and propagation of annotation mistakes over time.

Given the relatively tractable size of many phage genomes (3–550 kbp), many groups opt to manually curate the results of auto-annotation programs with additional information such as nucleotide sequence similarity matches, existence of operons, and synteny. The SEA-PHAGES program in particular, has pioneered the development of a set of guidelines for manually curating phage genomes that are auto-annotated by Glimmer and GeneMark to produce annotations of higher accuracy [25,26]. An additional benefit of manual curation of auto-annotated genomes is its high pedagogical value by providing students with a hands-on experience working with a genome. However, manual curation has several downsides, such as (1) introducing an element of subjectivity in decision making, which can potentially lead to an artificially fractured genomic landscape (different groups annotating similar phages may produce different annotations), (2) potentially significant time and labor costs if the number of phages to be annotated is large, (3) susceptibility to mistakes, which requires significant quality control to prevent, and (4) significant time investment in training annotators.

In this study, we present a genome annotation method developed in our lab that builds on the SEA-PHAGES guidelines in an effort to overcome these shortcomings. The method is designed to minimize subjectivity, increase speed and efficiency, and help minimize the occurrence mistakes. We validated the method by using it to annotate the genome of the coliphage Lambda, the most extensively studied phage [27], and the genome of mycobacteriophage Patience, the vast majority of whose genes have been verified through transcriptomics, mass-spec proteomics within infected cells, and identification of N-terminal peptides [28]. We then compared the annotations obtained using our method to the reference annotations in GenBank. We also compared the annotations obtained by our method to the annotations produced by Glimmer, the GeneMark family of programs, Prodigal, and PHANOTATE. We also tested our method and the above programs on a randomly generated DNA sequence, in which any genes identified are by definition false positives. Lastly, we show the pedagogical benefits of the method from assessment results from the phage genome annotation class taught at the University of Nevada, Las Vegas (UNLV).

## 2. Results

### 2.1. Gene Identification

The annotated genomes of phages Lambda (48.5 kbp) and Patience (70.5 kbp) were downloaded from NCBI GenBank (NC_001416.1 and NC_023691.1, respectively). These genomes were used to benchmark our manual curation method and compare it to the annotations produced by Glimmer, the GeneMark family of programs, Prodigal, and PHANOTATE. RAST and Prokka were omitted as they rely on Glimmer and Prodigal for gene calling, respectively. The GeneMark family includes the original GeneMark [16], host-trained GeneMark.hmm [17], GeneMark S [18], GeneMark with Heuristics [19], and the latest member of the family, GeneMark S2 [20]. While they may appear similar, each GeneMark algorithm is significantly different form the others and will produce a different annotation for the same genome [29].

With respect to gene identification, a positive (P) is a coding gene identified by one of the annotation methods (i.e., Glimmer, GeneMark family, Prodigal, or PHANOTATE, and our manual method); a true positive (TP) is a gene identified as coding by an annotation method that is coding in the reference annotated Lambda or Patience genome in GenBank; a false positive (FP) is defined as a gene identified as coding by an annotation method that is not coding in the reference genome; a false negative (FN) is an gene identified as non-coding by an annotation method but coding in the reference genome; and a true negative (TN) is an gene identified as non-coding by an annotation method that is also non-coding in the reference genome. We calculated the sensitivity (TP/(TP + FN)) and specificity (TN/(TN + FP)) of our manual curation method, Glimmer, the GeneMark family, Prodigal and PHANOTATE for Lambda and Patience. The results are shown in Table 1 and Table 2, respectively. The host-trained GeneMark.hmm algorithm [17] gave anomalous results with respect to the genome of phage Patience and is thus omitted from Table 2. This is likely due to the unusual nature of phage Patience, which appears to be a non-*Mycobacterium* phage that is in the process of migrating to *Mycobacteria* hosts [24]. Host-trained algorithms such as GeneMark.hmm thus have difficulty producing annotations for such phages.

The reference annotated genome of phage Lambda contains 73 coding genes and 545 non-coding ORFs longer than 75 base pairs (bp). All methods missed eight or more coding genes in the Lambda genome. This is due in part to the fact that reference Lambda genome contains at least two instances of genes within genes, which all annotation methods have difficulty with. Our manual curation method had the smallest number of false negatives and the highest sensitivity. Our method had marginally higher sensitivity than Glimmer, host-trained GeneMark.hmm and PHANOTATE, and significantly higher sensitivity than the other GeneMark programs and Prodigal. Of the GeneMark programs, host-trained GeneMark.hmm had the best performance, with the smallest number of false negatives. Glimmer had the best overall performance, Prodigal had the highest number of false negatives, and PHANOTATE had by far the highest number of false positives. Specificity is very high and roughly equal for all methods. This is due to the large (>500) number of true negatives (non-coding ORFS correctly identified as non-coding) and relatively small number of false positives. Thus, the increase in sensitivity of the manual method does not come at the cost of decreased specificity.

The genome of phage Patience contains 110 coding genes and 782 non-coding ORFs longer than 75 bp (not including tRNAs). The manual curation method missed only one coding gene in the reference Patience genome, and incorrectly identified only one non-coding ORF as coding, resulting in very high sensitivity and specificity (>99%). All auto-annotation programs had significantly more false negatives, with GeneMark S and PHANOTATE having the fewest. As with Lambda, PHANOTATE had the highest number of false positives, thus overall, GeneMark S had the best performance of the auto-annotation programs. Again, due to the large (>700) number of true negatives and small number of false positives, specificity was very high for all methods.

In analyzing the results of Table 1 and Table 2, the greater sensitivity of the manual curation method with respect to both phage genomes comes from two sources: The first is integrating the results of multiple auto-annotation programs. The various programs have entirely different algorithms, such that a gene missed by one program may be detected by another; integrating their results thus increases sensitivity. The other source of greater sensitivity is reliance on additional information, especially operons and coding potential, to detect short genes. Short genes (>150 bp) are especially difficult to detect for programs designed for prokaryotic genomes. There are several examples of short genes with coding potential or that are likely part of an operon (i.e., stop codon overlaps by 1, 4, or 8 bp with the stop codon of a gene on the same strand) that are detected by our method but are missed by one or more programs. Increased sensitivity often comes at the cost of decreased specificity. However, our method avoids false positives by using a threshold score (see Methods section) to decide whether an ORF is to be considered a coding gene. This reduces the likelihood of false positives by requiring multiple sources of supporting evidence in order to designate an ORF as coding.

### 2.2. Start Codon Identification

For identification of start codons, accuracy was calculated as the fraction of start codons identified during annotation that matched the start codons in the reference genome. Incorrect start codons resulted in a gene that was longer than in the reference genome, or a gene that was shorter than in the reference genome. The results for Lambda are shown in Table 3 and for Patience in Table 4. Host-trained GeneMark.hmm is omitted from Table 4.

For Lambda, the manual curation method is more accurate than any auto-annotation program, correctly identifying the start codons of 60 of 65 correctly identified coding genes (true positives). Prodigal had results similar to the manual curation method, while all other programs had roughly double the number of incorrectly identified start codons.

With respect to phage Patience, the manual curation method scored better than all programs. Of the programs, GeneMark and GeneMark S had the best performance, while PHANOTATE had the highest number of incorrect start codons in both Lambda and Patience. The manual curation method achieves higher accuracy for two reasons: First, by integrating the results of several programs, the manual method achieves a form of “consensus” among programs. While a particular program may identify a start codon incorrectly, it is unlikely that a large number of programs will do so. The second reason is the use of information such as operon existence and sequence similarity. Auto-annotation programs sometimes do not correctly identify start codons that result in an operon because these often have a low Shine-Dalgarno score; however, they are easily noticed when inspecting a genome manually. Sequence similarity matches reflect the consensus among annotators of similar phages and can provide insight into which start codon is more likely.

### 2.3. Susceptibility to False Positives in a Randomly Generated Nucleotide Sequence

To test the susceptibility of the various annotation methods to incorrectly identify ORFs known to be non-coding as coding (false positives), we generated a random, 40 kbp long nucleotide sequence and tested the manual curation method and auto-annotation programs on it. The sequence was generated in R using a random number generator with uniform probability distribution, and the specific sequence used in the analysis is included as Supplementary Material. The sequence has 628 ORFs longer than 75 bp. As all ORFs in the randomly generated sequence are non-coding, any ORFs identified as coding are false positives by definition. The results are shown in Table 5. Since there is no host to train GeneMark.hmm on, host-trained GeneMark.hmm is omitted from this analysis.

**Figure 2:**
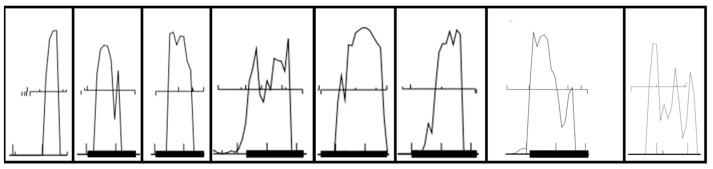
Coding potential of the eight open reading frames (ORFs) identified as coding in the randomly generated DNA sequence by our manual curation method. Six of these were identified as coding by GeneMark.

PHANOTATE and Glimmer identified a surprisingly large number of ORFs as coding, whereas the GeneMark programs, Prodigal, and the manual method identified a significantly smaller number. The manual method identifying two more ORFs as coding than the GeneMark family (with all four GeneMark programs having identical results). All of the ORFs identified as coding by the manual method possessed strong coding potential (Figure 2), but none possessed sequence similarity matches. Six of the ORFs identified as coding by the manual method were also identified by the GeneMark programs (which follows since the manual curation method partially relies on auto-annotation programs). These results suggest that PHANOTATE and Glimmer are prone to generating false positives; that coding potential, while a very useful indicator of whether a gene is coding (positive signal), is also prone to generating false positives; and that lack of sequence similarity matches, while not a reliable positive signal (due to false positives in the record), is a strong negative signal.

### 2.4. Pedagogical Benefits

**Figure 3:**
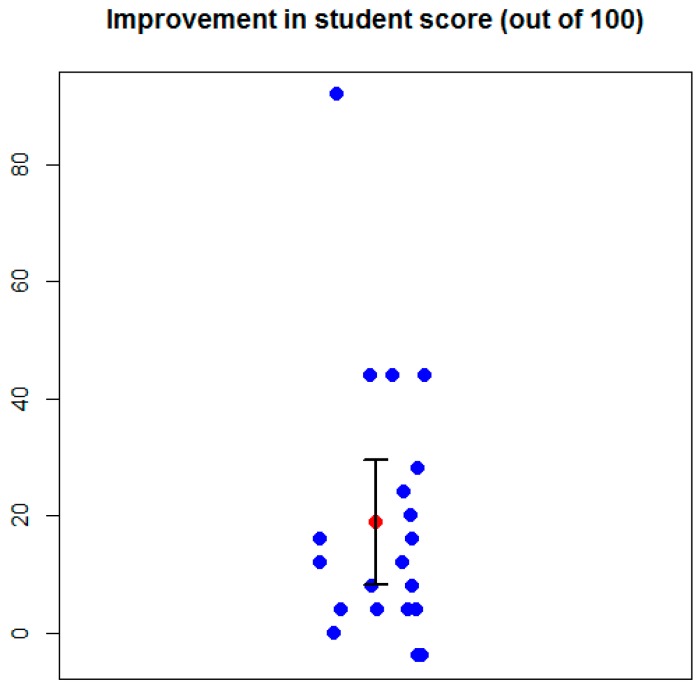
Change in student score on the assessment from the start to the conclusion of the genome annotation course. Each blue point represents a student, the red point represents the mean, and the 95% confidence interval is shown in black.

Students enrolled in UNLV’s phage genome annotation course use the method presented in this study to annotate the genomes of phages they have isolated. At the start of the semester, students enrolled in the class complete a multiple choice assessment covering fundamental concepts in molecular biology and microbiology. There are two versions of the assessment, A and B, with the same questions albeit worded differently in each version. At the end of the semester, students complete the assessment again, with students that completed version A at the beginning of the semester completing version B at the end, and vice versa. The two versions of the assessment are included as Supplementary Material. The change in the students’ scores on the assessment are shown in Figure 3.

The average change in the students’ scores was 20% with standard deviation 23. The *p*-value of the paired *t*-test is 0.0016, with the 95% confidence interval between –29.5 and –8.1. Of the 20 students in the class, 11 students improved by 10% or more, seven improved by 20% or more, and one student (who had no biology background prior to taking this course) improved by more than 90%. Three of 20 students did not improve, with the score of two students slightly decreasing, but these students had scored very high on the assessment at the beginning of the semester. As the students are not issued a textbook or lectures on molecular genetics and microbiology, these results tentatively suggest that the improvement in student scores comes from annotating phage genomes.

## 3. Discussion

In this study, we present a method for manually curating annotated phage genomes that is more sensitive than purely automated methods while designed to minimize the occurrence of mistakes and subjectivity in decision-making. By separating the criteria used to evaluate genes and start codons, and assigning numerical values for each criterion, the method reduces subjectivity in decision-making (see Methods section). We have designed the method to be as labor efficient as possible, and estimate an experienced annotator using the method could annotate a typical phage genome (40–50 kbp) in one or two days. The method is designed to be user-friendly and amenable to instruction to undergraduate students. The method is currently being used to annotate phage genomes isolated by students at our institution. The pedagogical benefits of training students in the use of the method were shown to be statistically significant, as evidenced by an assessment conducted at our institution.

One of the main challenges of devising annotation methods for phage genomes is identifying reference, experimentally validated genomes to benchmark a method’s accuracy. To the best of our knowledge, the best choices for reference genomes are the genome of *Escherichia coli* phage Lambda, as it is the most extensively studied bacteriophage genome [27], and the genome of mycobacteriophage Patience, as the vast majority of its genes have been validated through transcriptomics [28]. We thus used these two genomes to benchmark the performance of our method and compare it to widely used annotation programs such as Glimmer, the GeneMark family of programs, Prodigal, and the recently-developed program PHANOTATE.

Our method showed slightly to moderately higher sensitivity with respect to gene identification in comparison to all auto-annotation programs, without a decrease in specificity. The manual curation method was also more accurate in identifying start codons. The manual curation method was consistent across the two phage genomes, producing similar results for Lambda and Patience. The auto-annotation programs had more divergent results, with some performing significantly better on Patience (GeneMark S, Prodigal) and some performing better on Lambda (Glimmer, host-trained GeneMark.hmm). Of the auto-annotation programs, GeneMark S seemed to have the best performance overall, while no program performed better or worse in all categories compared to the others. Host-trained GeneMark.hmm had very poor results with respect to phage Patience. This is due to the unusual nature of Patience, and does not reflect generally on host-trained hidden Markov models. However, it does signify that host-trained algorithms may not always be appropriate for use on certain phage genomes. Surprisingly, the program PHANOTATE, specifically designed for phage genomes, did not outperform the others.

The greater accuracy of the manual curation method relative to individual auto-annotation programs is largely due to two reasons: First, integrating the results of multiple auto-annotation programs to achieve consensus, and second, relying on additional information such as operons, coding potential, and sequence similarity. By integrating the results of multiple programs, each of which uses a different algorithm from the others, the manual curation method may detect genes missed by an individual program and achieve “consensus” among the programs when choosing a start codon. Similarly, by relying on additional information, the manual curation method may detect genes and start codons that are particularly difficult for auto-annotation programs to detect (e.g., short genes and start codons with low Shine-Dalgarno score). Higher sensitivity usually comes at the cost of lower specificity (more false positives), however, by requiring multiple sources of information and setting a threshold for decision-making, the manual curation method largely avoids the low specificity trap.

This study represents a first step in devising rigorous manual curation methods for phage genome annotations, as well the first step in establishing the accuracy of various auto-annotation programs with respect to phage genomes. While the results of the study are encouraging, the method used herein needs to be applied to more experimentally validated phage genomes for the conclusion of this study to be more generally applicable. Lack of experimentally validated phage genome is a long-standing challenge in the area of phage genomics, and more work needs to be done in this area to put phage genome annotations on a firmer footing.

## 4. Materials and Methods

The annotation method presented here is based on guidelines developed and described by Pope et al. [25,26]. The innovation of our method is to put these guidelines in more rigorous form, by clearly delineating the various sources of information and devising a scheme to quantify and integrate them so as to reduce subjectivity in decision-making. There are two main steps: Gene identification, and start codon identification. The third component of genome annotation, putative function assignment, is not covered in this publication.

### 4.1. Gene Identification

The first step of genome annotation is identifying genes. Putative genes are assigned a score that determines whether they are identified as coding or discarded based on five criteria: (a) Number of auto-annotation programs that identified the gene as coding (Glimmer, the GeneMark family of programs, Prodigal, PHANOTATE); (b) existence of coding potential as predicted by GeneMark S (i.e., the posterior decoding of the hidden Markov model); (c) existence of statistically significant nucleotide sequence similarity matches; (d) existence of overlaps with other putative genes, including whether the putative gene is part of an operon, and (e) ORF length. The coordinates and reading frame of genes to be evaluated by the method are entered in a spreadsheet, with a column for each criterion: “Programs,” “Coding potential,” “Sequence similarity matches,” “Overlap,” and “Length”.

#### 4.1.1. Auto-Annotation Program Calls

**Figure 4:**
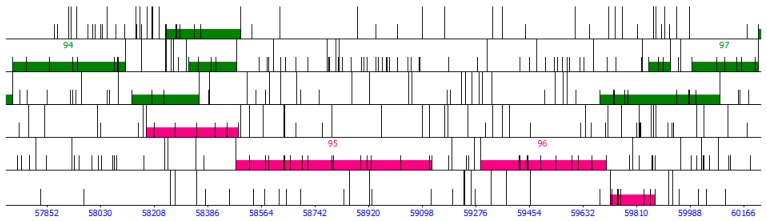
Examples of program-identified genes and user-identified putative genes in DNA Master. Each row corresponds to a reading frame; the forward (left to right) reading frames in the top three rows; the reverse (right to left) reading frames in the bottom three rows. Half height bars represent start codons; full height bars represent stop codons. Highlighted bars represent putative genes, with green for forward genes and magenta for reverse genes. Numbered genes indicate genes identified by Glimmer or GeneMark; genes without numbers indicate putative user-identified genes not identified by Glimmer and GeneMark. There are four putative user-identified genes located in the gap between gene 94 and gene 95 (three forward, one reverse) and three between gene 96 and 97 (two forward, one reverse).

On first pass, the genome is auto-annotated with Glimmer [15] and GeneMark [16] using the program DNA Master (http://cobamide2.bio.pitt.edu/computer.htm), which serves as the primary genome viewing browser. Detailed instructions on how to use DNA Master are given in [25,26]. DNA Master also includes the program ARAGORN, which detects tRNA genes with a high degree of accuracy [30]. The auto-annotated genome in DNA Master is then searched for coding gaps (i.e., regions where neither Glimmer nor GeneMark identified genes). ORFs longer than 75 bp that are located in coding gaps are noted and their coordinates (5′ end, 3′ end, reading frame) entered into a spreadsheet. These are potential false negatives (i.e., missed by Glimmer and GeneMark). We choose a threshold of 75 bp as phage proteins as short as 27 amino acids have been found experimentally [25]. Two coding gaps that are filled with putative, user-identified genes are shown in Figure 4.

The genome is then auto-annotated outside of DNA Master with host-trained GeneMark [17], GeneMark S [18], Heuristic GeneMark [19], and GeneMark S2 [20] via their web servers (www.exon.gatech.edu), and also with Prodigal (https://github.com/hyattpd/Prodigal) and PHANOTATE (https://github.com/deprekate/PHANOTATE) by downloading the latter two and running them locally. We paste the output from all the programs in a single spreadsheet, which is then used to determine how many programs identify a gene; this is also very useful when assigning start codons. The number of programs that identify a putative gene as coding is entered into that gene’s “Programs” column (can range from 0 to 8).

#### 4.1.2. Coding Potential

**Figure 5:**
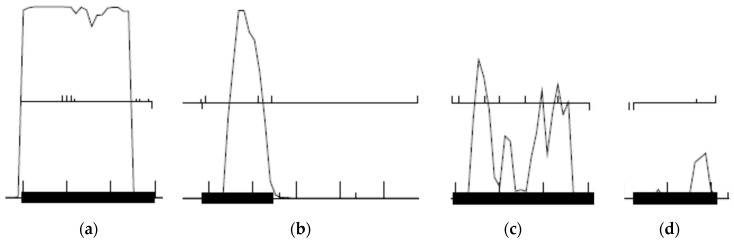
Examples of coding potential predicted by GeneMark S. Upward ticks represent start codons, downward ticks represent stop codons, and horizontal lines between ticks indicate ORFs. (**a**) The first putative gene on the left has high and sustained coding potential and is scored 3 points; (**b**) the second putative gene from the left has high but not sustained coding potential and is scored 2 points; (**c**) the third putative gene from the left has low but sustained coding potential and is scored 1 point; (**d**) the last putative gene on the right has coding potential that is low and not sustained and is thus scored 0 points.

Coding potential (the posterior decoding of a hidden Markov model for gene prediction) is among the strongest evidence for gene prediction [26], and putative genes with strong coding potential are unlikely to be false positives. Each putative gene is scored from 0 to 3 based on its coding potential as predicted by GeneMark S (Figure 5). A putative gene with high (>0.75) and sustained (covers >50% of the ORF) coding potential is scored 3; a putative gene with high but not sustained coding potential scored 2; a putative with low but sustained coding potential scored 1; and a putative gene with neither high nor sustained coding potential scored 0. Examples of each type of coding potential score are shown in Figure 5. The coding potential score is entered in the “Coding potential” column for each putative gene’s spreadsheet entry.

#### 4.1.3. Sequence Similarity Matches

Statistically significant amino acid sequence similarity matches are an indicator of whether a gene with similar sequence has been identified as coding by other researchers in the field. Putative genes with significant sequence similarity matches to known proteins, especially to proteins with known function, are unlikely to be false positives. Sequence similarity matches are identified by batch searches of NCBI’s non-redundant (nr) database with pBLAST [31], and searches of Pfam and Interpro with HMMer [32]. Putative genes are scored 1 point if they have at least one sequence similarity match with E-value less than E-10 (this threshold can be adjusted); 2 points if they have a sequence similarity match with E-value between E-20 and E-50; and 3 points if they have a sequence similarity match with E-value less than E-50. Putative genes are scored an additional point if one of the sequence similarity matches is to a protein with known function, as opposed to a “hypothetical protein.” The sequence similarity match score (range from 0 to 4) is entered in the “Sequence similarity” column for each putative gene’s spreadsheet entry.

#### 4.1.4. Overlap and Operons

**Figure 6:**
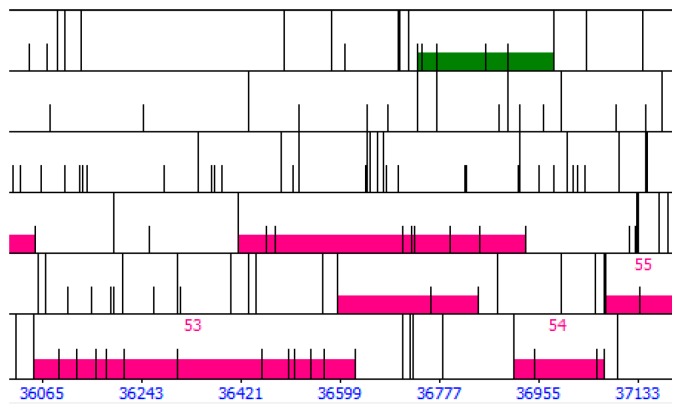
Examples of overlap as visualized in DNA Master. There are three putative user-identified genes located in the gap between gene 53 and gene 54 (two reverse, one forward). The putative gene in reverse frame 1 (row 4) overlaps with gene 53 and gene 54; the putative gene in reverse frame 2 (row 5) overlaps with gene 53; the putative gene in forward frame 1 (row 1) overlaps with gene 54. The putative gene in reverse frame 1 appears to form an operon with gene 54 (4 bp overlap). There is a gap of more than 50 bp between gene 53 and the putative forward gene, enough to make room for their promoters. The three putative genes overlap with one another, but for each gene, only the overlap with gene 53 and gene 54 is counted (because these are strong gene calls).

Phage genes seldom overlap by more than 30 bp, and thus putative genes that significantly overlap with strong gene calls are more likely to be false positives [26]. We thus penalize putative genes with significant overlap with strong gene calls (i.e., genes identified by both Glimmer and GeneMark, as such genes are unlikely to be false positives). Examples of overlap are shown in Figure 6. Overlap less than 10 bp are not penalized; overlap between 10 and 40 bp is penalized 1 point; overlap between 40 and 70 bp penalized 2 points; overlap between 70 and 100 bp penalized 3 points, and an overlap of more than 100 bp penalized 4 points. This score is entered in the “Overlap” column for each putative gene’s entry in the spreadsheet. A forward gene downstream of a reverse gene represents a special case, as this requires at least a 50 bp gap between the two genes to make room for their promoters [26].

It should be noted that overlap is only scored for putative genes when these overlap with genes previously identified by both Glimmer and GeneMark, as such genes are unlikely to be false positives. In the case of multiple overlapping putative genes within a coding gap, overlap is not scored, as it is at this stage unknown which putative genes will be kept as coding, if any.

Overlaps of 1, 4, or 8 bp present special cases, as genes with such overlaps are often part of an operon [33]. Operons are a common occurrence in phage genomes. As it is unlikely that false positives would be part of an operon, we award 1 point to a putative gene that has a start codon that overlaps with the stop codon of another gene by 1, 4, or 8 bp.

The “overlap” score can range from –4 to 1, and is entered in the “Overlap” column for each putative gene’s spreadsheet entry. Examples of overlapping putative genes are shown in Figure 6.

#### 4.1.5. ORF Length

Non-coding ORFs tend to be short (<200 bp), while genes in very long ORFs are less likely to be false positives. We thus score putative genes according to their length. Putative genes with ORFs shorter than 200 bp but longer than 150 bp are penalized 1 point; ORFs shorter than 150 bp but longer than 120 bp are penalized 2 points; ORFs shorter than 120 bp but longer than 90 bp are penalized 3 points; and ORFs shorter than 90 bp are penalized 4 points. The length score (range –4 to 0) is entered in the “Length” column for each ORF’s entry in the spreadsheet. Length will depend on the start codon chosen, and sometimes there may be cases where choosing a start codon that produces a shorter ORF may mitigate overlap with another gene.

#### 4.1.6. Checking for False Positives

Once all potential false negatives (genes missed by Glimmer and GeneMark) are scored as described above, we do the same for potential false positives. Potential false positives are genes identified as coding by only one program (e.g., Glimmer only, GeneMark only, etc.), or genes identified by more than one program but with very short ORFs (shorter than 120 bp). Potential false positives are entered in the spreadsheet and evaluated according to the five criteria described above, in exactly the same manner as the potential false negatives. Theoretically all putative genes, both identified by programs and identified by users should be evaluated this way, but in practice genes identified by two or more programs are extremely unlikely to be false positives, and thus only user-identified genes and genes identified by only one program should be evaluated to save time and effort.

#### 4.1.7. Decision-Making for Gene Identification

For each putative gene to be evaluated, the scores in each column are summed and a decision is made whether to keep the putative gene as coding or discard it as non-coding. An example of what the spreadsheet should look like is given in Table 6. We suggest putative genes with a score of 3 or greater be kept, and 0 or less discarded. This is so that genes must have either program calls, coding potential, or sequence similarity matches in order to be kept, and simply having a long ORF and not overlapping is not sufficient reason for a putative gene to be kept as coding. Scores of 1 and 2 are borderline cases to be decided on a case-by-case basis.

### 4.2. Start Codon Identification

Most phage genes contain more than one start codon in their nucleotide sequence. For annotation purposes it is necessary to pick one start codon as the annotated gene start. Start codons are scored on six criteria, in the following order of importance: (1) whether a start codon produces an ORF that includes all coding potential; (2) whether a start codon produces an ORF that overlaps with other genes to form an operon; (3) the number of auto-annotation programs that pick that start codon; (4) whether the start codon matches with the start of highly similar proteins; (5) Shine-Dalgarno (SD) score; (6) the resultant ORF length. For each gene, an entry is created in the spreadsheet, with the candidate start codons in the first column, and a column for each criterion: “Coding potential,” “Overlap/Gap,” “Programs,” “Sequence similarity,” “SD score,” and “Length.”

#### 4.2.1. Coding Potential

**Figure 7:**
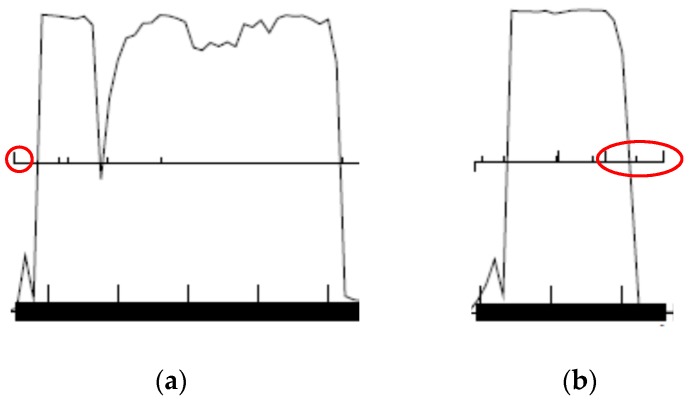
(**a**) The gene on the left, transcribed from left to right, has six start codons (upward ticks above the midline in the figure). Of these, only the leftmost one, circled in red, includes all of the gene’s coding potential, as it is located upstream of the coding potential plateau; (**b**) the gene on the right, transcribed from right to left, has eight start codons. Of these, the rightmost three, circled in red, include all the coding potential, while the remaining five do not. When deciding, only the plateau in coding potential counts, not the slope.

It is important that a start codon includes all of a gene’s coding potential as shown in Figure 7. For each start codons a “yes” or “no” is entered in the “Coding potential” column based on whether the start codon includes all of a gene’s coding potential. Start codons that fail to include all of a gene’s coding potential are the first to be eliminated.

#### 4.2.2. Overlap or Gap

**Figure 8:**
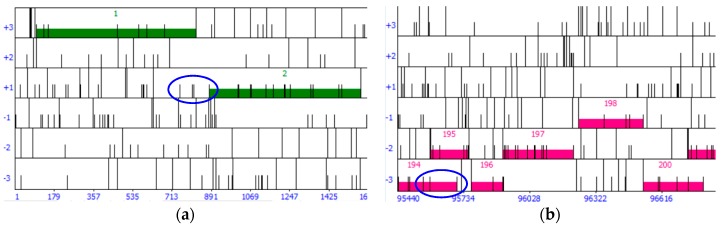
(**a**) For forward genes, such as gene 2, the overlap of each start codon considered (circled in blue) is calculated by subtracting each start codon from the stop codon of gene 1; (**b**) for reverse genes, such as gene 194, the overlap of each start codon evaluated (circled in blue) is calculated by subtracting the stop codon of gene 195 from each start codon of gene 194. To save time and effort, only start codons that produce a long ORF are evaluated, e.g., the majority of gene 2’s start codons in panel (a) are not evaluated.

The overlap or gap of each start codon with the stop codon of the upstream or downstream gene is calculated and entered in the “overlap/gap” column. For genes transcribed left to right (forward genes), the start codon is subtracted from the stop codon of the gene upstream to calculate overlap (Figure 8). For genes transcribed right to left (reverse genes), the stop codon of the gene downstream is subtracted from the start codon (Figure 8). This produces a positive number in the case of overlap, and a negative number in case of a gap. For overlaps, 1 must be added to the subtraction result, and for gaps, 1 must be subtracted. Start codons that produce an unusually large overlap (e.g., over 100 bp) when there exist start codons that include all coding potential are not considered. For the special case of a forward gene followed by a reverse gene, a table is created in which the starts of the forward gene are the rows and the starts of the reverse genes the columns. In each cell, the gap or overlap is calculated by subtracting the start codon of the reverse gene from the start codon of the forward gene and adding 50 (to account for the promoters) [26].

#### 4.2.3. Auto-Annotation Program Calls

All auto-annotation programs identify a start codon for every gene they identify. For each start codon, the number of programs that identify it as the gene’s start is entered in the “Programs” column.

#### 4.2.4. Sequence Similarity Matches

For each start codon, the number of sequence similarity matches with an E-value less than 1E-10 (pBLAST and/or HMMer) that use that start codon is entered in the “Sequence similarity matches” column.

#### 4.2.5. Shine-Dalgarno (SD) Score

The Shine-Dalgarno score of each start codon is calculated using DNA Master and entered in the “SD score” column. The settings are “Kibler6” for the SD scoring matrix and “Karlin Medium” for the spacing matrix [25,26]. The SD score is the number given in the “Final score” column. This number is always negative, and a smaller absolute value (i.e., closer to 0) is considered a better score. If a start codon is likely to indicate the existence of an operon (1, 4, or 8 bp overlap), the SD score is considered irrelevant and is not entered.

#### 4.2.6. Length

If two start codons are equal with respect to all of the above criteria, ORF length may be used as a tie-breaker, with the start codon that produces the longest ORF without overlap of more than 10 bp chosen.

#### 4.2.7. Decision-Making for Start Codons

An example of a start codon annotation for a gene is shown in Table 7. The start codons are sorted by length (i.e., the start that produces the longest ORF is in the first row), and the start codon that is the best choice according to our method (605) is shown in boldface. The start codon at 626 is the first to be eliminated because it does not include all the coding potential, despite the higher number of program calls and better SD score. The start codon at 442 is then eliminated because of its large overlap (128 bp). The start codon at 578 is the next to be eliminated because it has no program calls. The choice is then between 605 and 587, with 605 chosen over 587 on the grounds of two programs choosing it versus one for 587 (and despite 587 having more sequence similarity matches).

In some cases, it is possible to save time and effort by skipping the full analysis. This is the case when a gene has only one start codon (or only one start codon that does not result in an excessively short ORF), or when the start codon that produces the longest possible ORF is chosen by all programs (as it is impossible for shorter codons to score better on coding potential).

## Figures and Tables

**Table 1:** Results of gene identification in the genome of phage Lambda. Sensitivity and specificity are rounded to the nearest 0.5%.

Annotation Method	Positives	True Positives	False Positives	False Negatives	True Negatives	Sensitivity	Specificity
**Glimmer**	67	63	4	10	541	86.5%	99.5%
**GeneMark**	61	58	3	15	542	79.5%	99.5%
**GeneMark hmm**	66	62	4	11	541	85%	99.5%
**GeneMark S**	61	58	3	15	542	79.5%	99.5%
**Heuristic GeneMark**	61	58	3	15	542	79.5%	99.5%
**GeneMark S2**	60	58	2	15	543	79.5%	99.5%
**Prodigal**	61	55	6	18	539	75.5%	99%
**PHANOTATE**	88	62	26	11	519	85%	95%
**Manual**	**69**	**65**	**4**	**8**	**541**	**89%**	**99%**

**Table 2:** Results of gene identification in the genome of mycobacteriophage Patience. Host-trained GeneMark.hmm is omitted due to its anomalous results for this phage.

Annotation Method	Positives	True Positives	False Positives	False Negatives	True Negatives	Sensitivity	Specificity
**Glimmer**	104	99	5	11	777	90%	99.5%
**GeneMark**	90	90	0	20	782	82%	100%
**GeneMark S**	105	105	0	5	782	95.5%	100%
**Heuristic GeneMark**	101	100	1	10	781	91%	99.9%
**GeneMark S2**	94	93	1	17	781	84.5%	99.9%
**Prodigal**	105	101	4	9	778	92%	99.5%
**PHANOTATE**	120	105	15	5	767	95.5%	98%
**Manual**	**110**	**109**	**1**	**1**	**781**	**99%**	**99.9%**

**Table 3:** Results of start codon identification in the genome of phage Lambda.

Annotation Method	True Positives	Start Codons Called Long	Start Codons Called Short	Total Number of Incorrect Start Codons	% Correct Start Codons
**Glimmer**	63	6	5	11	82.5%
**GeneMark**	58	4	7	11	81%
**GeneMark hmm**	62	1	8	9	85.5%
**GeneMark S**	58	6	5	11	81%
**Heuristic GeneMark**	58	6	5	11	81%
**GeneMark S2**	58	3	7	10	83%
**Prodigal**	55	3	3	6	89%
**PHANOTATE**	62	7	6	13	79%
**Manual**	**65**	**1**	**4**	**5**	**92.5%**

**Table 4:** Results of start codon identification in the genome of mycobacteriophage Patience. Host-trained GeneMark.hmm is omitted due to its anomalous results for this phage.

Annotation Method	True Positives	Start Codons Called Long	Start Codons Called Short	Total Number of Incorrect Start Codons	% Correct Start Codons
**Glimmer**	99	3	11	14	86%
**GeneMark**	90	4	4	8	91%
**GeneMark S**	105	4	4	8	92.5%
**Heuristic GeneMark**	100	9	6	15	85%
**GeneMark S2**	93	9	7	16	83%
**Prodigal**	101	7	6	13	87%
**PHANOTATE**	105	8	13	21	80%
**Manual**	**109**	**4**	**3**	**7**	**93.5%**

**Table 5:** Results of annotation of a randomly generated nucleotide sequence by the manual curation method and auto-annotation programs.

Annotation Method	False Positives	True Negatives	Specificity
**Glimmer**	47	581	92.5%
**GeneMark**	6	622	99%
**GeneMark S**	6	622	99%
**Heuristic GeneMark**	6	622	99%
**GeneMark S2**	6	622	99%
**Prodigal**	11	617	98%
**PHANOTATE**	203	425	68%
**Manual**	**8**	**620**	**99%**

**Table 6:** Typical entries for putative genes with evaluation result.

Reading Frame	5′ End	3′ End	Programs	Coding Potential	Sequence Similarity	Overlap	Length	Points	Results
F1	373	495	0	0	0.0018	0	204	0	Delete
R2	2189	2391	1	2	<E-50	43 bp	153	3	Add
R1	50,061	50,159	4	3	E-13	4 bp	99	6	Add

**Table 7:** An example of a start codon annotation according to our method.

Start Coordinate	Programs	Coding Potential	Overlap	No. of Sequence Similarity Matches	SD Score
442	0	Yes	128 bp	0	−5.9
578	0	Yes	0 bp	1	−5
587	1	Yes	0 bp	4	−6.5
**605**	2	Yes	0 bp	2	−4.6
626	3	No	0 bp	1	−3.8

## Supplementary Materials

Supplementary materials can be found at https://www.mdpi.com/1422-0067/20/14/3391/s1. The following supplementary materials are included: (1) Version A and Version B of the assessment issued to the students enrolled in the University of Nevada Las Vegas (UNLV)’s phage genome annotation class (BIOL 217). (2) The randomly generated 40 kbp DNA sequence used in Section 2.3 of the results.

## Abbreviations

SEA-PHAGES	Science Education Alliance-Phage Hunters Advancing Genomics and Evolutionary Sciences
kbp	Kilo base pairs
UNLV	University of Nevada Las Vegas
bp	Base pairs
TP	True positive
FP	False positive
TN	True negative
FN	False negative
ORF	Open reading frame
SD	Shine-Dalgarno
