# Supplementary material for: A Method for Improving the Accuracy and Efficiency of Bacteriophage Genome Annotation
Source: Int J Mol Sci. 2019 Jul 10;20(14):3391. doi: 10.3390/ijms20143391 (PMC6678273; doi:10.3390/ijms20143391)
Supplement: Supplementary file 1 [file ijms-20-03391-s001.zip › ijms-527198-supp/Assessment version A.pdf]

BIOL 209X Assessment

1. A gene is 90 base pairs long. The protein coded by this gene is __ amino acids long.
   - A) 20
   - B) 30
   - C) 45
   - D) 60
   - E) 90

2. A mutation is…
   - A) The silencing of coding sections of DNA
   - B) The production of amino acids from nucleotides
   - C) The production of DNA from mRNA
   - D) The destruction of DNA by enzymes
   - E) Any change to an organism's DNA sequence

3. The transmission of genes from peer to peer in a population is known as…
   - A) Translocation
   - B) Transmigration
   - C) Vertical gene transfer
   - D) Horizontal gene transfer

4. What is the correct flow of information?
   - A) mRNA → DNA → Protein
   - B) DNA → Protein → mRNA
   - C) Protein → DNA → mRNA
   - D) DNA → mRNA → protein
   - E) mRNA → Protein → DNA

5. Viruses do NOT possess…
   - A) Peptidoglycan cell wall
   - B) Mitochondria
   - C) Ribosomes
   - D) Nucleus
   - E) All of the above, viruses do not have any organelles

6. Amino acids are the building blocks of ______.
   - A) Nucleotides
   - B) Monosaccharides
   - C) Lipids
   - D) Proteins

7. A ______ is a segment of ____ that contains the information for making a ______.
   A) Protein; DNA; Gene.
   B) Gene; Amino Acids; Protein
   C) Protein; Amino Acids; Gene
   D) Chromosome; DNA; Gene.
   E) Gene; DNA; Protein.

8. The process of making polypeptides from mRNA is known as…
   A) Translation
   B) Mutation
   C) Replication
   D) Transcription
   E) Transduction

9. If a sequence of one DNA strand is 5'-TAGC-3', then the sequence of the opposite strands is…
   A) 5'-ACTG-3'
   B) 3'-ATCG-5'
   C) 5'-TAGC-3'
   D) 3'-ATGC-5'
   E) 5'-ATGC-3'

10. A genome is …
   A) All of the genes of an organism
   B) All of the DNA in an organism
   C) All of the proteins in an organism
   D) All the amino acids in an organism
   E) All of the RNA in an organism

11. Double stranded DNA has _____ possible reading frames
   A) 2
   B) 3
   C) 4
   D) 5
   E) 6

12. A DNA strand is always read in the…
   A) 1’ to 2’ direction
   B) 2’ to 1’ direction
   C) 3’ to 5’ direction
   D) 5’ to 3’ direction
   E) 1’ to 3’ direction

13. A stretch of DNA between a Start and a Stop codon is known as a….
   A) Gene
   B) Reading frame
   C) Open reading frame
   D) Non-coding region
   E) Intron

14. The enzyme responsible for DNA replication is…
   A) DNA polymerase
   B) DNase
   C) RNase
   D) RNA polymerase
   E) DNA transcriptase

15. During translation, amino acids are provided by
   A) DNA
   B) mRNA
   C) tRNA
   D) rRNA
   E) mtDNA

16. Gene expression is…
   A) The production of a protein that a gene codes for
   B) The replication of a gene
   C) A change in the DNA sequence of a gene
   D) The production of tRNA from mRNA
   E) The lengthening of a gene

17. According to the rules of DNA, which of the following are equal in a genome?
   A) A+T=G+C
   B) A=T and C=G
   C) A=G and C=T
   D) A=C and G=T

18. Lysogeny is the process by which phages…
    A) Destroy their host and escape
    B) Integrate their DNA into the host’s DNA
    C) Enter their host
    D) Carry DNA from one bacterial cell to another
    E) Degrade the host’s DNA

19. Phenotype is the …
    A) Physical result of a genetic trait
    B) DNA sequence of a genetic trait
    C) mRNA sequence of a genetic trait
    D) Length of an organism’s genome
    E) Number of offspring of an organism

20. All phages possess a…
    A) Capsule
    B) Peptidoglycan cell wall
    C) Capsid
    D) Nucleus
    E) Mitochondria

21. Transduction is the process by which…
    A) Phage genomes replicate inside their host
    B) Phages enter their host
    C) Phages escape from their host
    D) Bacteria share plasmids with one another
    E) Phages transmit genetic information from on bacterium to another

22. The process of identifying genes, start codons, and assigning function to genes in an organism’s genome is known as
    A) Genome sequencing
    B) Genome assembly
    C) Genome annotation
    D) Genome transcription
    E) Genome translation

23. A phage that is *virulent* is one that…
   A) Integrates its DNA in the host’s DNA
   B) Reproduces inside its host and lyses its host
   C) Is able to attach to its host
   D) Is unable to attach to its host
   E) Is unable to escape from its host

24. Using the genetic code given below, what will be the amino acid sequence produced by the following mRNA sequence: 5'-AUG CGC UCG CUA UAU UAA-3'

	Second Position								
	U		C		A		G		
**First Position**									**Third Position**
U	UUU	Phe	UCU	Ser	UAU	Tyr	UGU	Cys	U
	UUC		UCC		UAC		UGC		C
	UUA	Leu	UCA		UAA	Stop	UGA	Stop	A
	UUG		UCG		UAG	Stop	UGG	Trp	G
C	CUU	Leu	CCU	Pro	CAU	His	CGU	Arg	U
	CUC		CCC		CAC		CGC		C
	CUA		CCA		CAA	Gin	CGA		A
	CUG		CCG		CAG		CGG		G
A	AUU	Ile	ACU	Thr	AAU	Asn	AGU	Ser	U
	AUC		ACC		AAC		AGC		C
	AUA		ACA		AAA	Lys	AGA	Arg	A
	AUG	Met (start)	ACG		AAG		AGG		G
G	GUU	Val	GCU	Ala	GAU	Asp	GGU	Gly	U
	GUC		GCC		GAC		GGC		C
	GUA		GCA		GAA	Glu	GGA		A
	GUG		GCG		GAG		GGG		G

   A) N-Met-Arg-Ser-Leu-Glu-C
   B) N-Met-Arg-Ser-Leu-Tyr-C
   C) N-Met-Arg-Ser-Pro-Gly-C
   D) N-Met-Arg-Ser-Leu-Trp-C
   E) N-Met-Cys-Ser-Leu-Tyr-C

25. A mutation that does NOT result in a change in the amino acid of a polypeptide is a _______ mutation.
   A. Point
   B. Frameshift
   C. Silent
   D. Missense
   E. Nonsense
