# Supplementary material for: A Method for Improving the Accuracy and Efficiency of Bacteriophage Genome Annotation
Source: Int J Mol Sci. 2019 Jul 10;20(14):3391. doi: 10.3390/ijms20143391 (PMC6678273; doi:10.3390/ijms20143391)
Supplement: Supplementary file 1 [file ijms-20-03391-s001.zip › ijms-527198-supp/Assessment version B.pdf]

BIOL 209X Assessment

1. How many DNA bases does it take to code for one amino acid?
   A) 1
   B) 2
   C) 3
   D) 4
   E) 6

2. Any change to an organism's original DNA sequence is a case of …
   A) Translocation
   B) Transformation
   C) Translation
   D) Mutation
   E) Transcription

3. What is horizontal gene transfer?
   A) The transmission of genes from parents to offspring
   B) The transmission of genes from one chromosome to another
   C) The transmission of genes from peer to peer in a population
   D) The transfer of genes from the sperm to the egg

4. The central dogma of molecular biology is…
   A) mRNA → DNA → Protein
   B) DNA → Protein → mRNA
   C) DNA → mRNA → protein
   D) Protein → DNA → mRNA
   E) mRNA → Protein → DNA

5. Which of the following organelles do viruses possess?
   A) Plasma membrane
   B) Mitochondria
   C) Ribosomes
   D) Nucleus
   E) None of the above, viruses do not have any organelles

6. The building blocks of proteins are…
   A) Nucleotides
   B) Monosaccharides
   C) Lipids
   D) Amino Acids

7. What is the relationship between genes and proteins?
   A) Proteins store the information needed for making genes.
   B) Genes contain the information for making ATP; Proteins contain the information for making enzymes.
   C) Genes determine an organism's phenotype; Proteins determine an organism's genotype.
   D) Genes are the products of protein synthesis.
   E) Genes are segments of DNA that contain the information for synthesizing proteins.

8. The process of making mRNA from DNA is known as…
   A) Translation
   B) Mutation
   C) Replication
   D) Transcription
   E) Transduction

9. If a sequence of a DNA strand is 5'-TAGC-3', then the mRNA sequence synthesized from it is…
   A) UAGC
   B) ATCG
   C) TAGC
   D) ATGC
   E) AUCG

10. The totality of an organism's DNA is known as its…
    A) Phenome
    B) Genome
    C) Chromosome
    D) Proteome
    E) Genotype

11. How many reading frames does double stranded DNA possess?
    A) 2
    B) 3
    C) 4
    D) 5
    E) 6

12. A DNA strand is always read in the…
   A) 1' to 2' direction
   B) 3' to 5' direction
   C) 5' to 3' direction
   D) 1' to 3' direction
   E) 2' to 1' direction

13. An open reading frame is a…
   A) Gene
   B) Stretch of DNA between a Start and a Stop codon
   C) Stretch of DNA between two Start codons
   D) Stretch of DNA between a Stop and a Start codon
   E) Non-coding stretch of DNA

14. The enzyme responsible for DNA transcription is…
   A) DNA polymerase
   B) DNase
   C) RNase
   D) RNA polymerase
   E) DNA transcriptase

15. The function of tRNA is …
   A) To mediate between DNA and polypeptides
   B) To transport mRNA to the ribosomes
   C) To provide amino acids for the translation of mRNA into a polypeptide
   D) The main component of ribosomes
   E) To facilitate the replication of DNA

16. When a gene is "turned on" and the protein it codes for is produced by the cell, we say the gene is…
   A) Expressed
   B) Activated
   C) Mutated
   D) Translated
   E) Silenced

17. According to the rules of DNA, which of the following are NOT equal in a genome?
   A) A+T=G+C
   B) A+G=C+T
   C) A+C=G+T
   D) A=T and G=C

18. The process by which phages escape and destroy their host is known as…
    A) Lysis
    B) Lysogeny
    C) Extrusion
    D) Translocation
    E) Transduction

19. An organism’s physical appearance is it’s…
    A) genotype
    B) karyotype
    C) haplotype
    D) morphotype
    E) phenotype

20. All bacteria possess a…
    A) Capsule
    B) Peptidoglycan cell wall
    C) Capsid
    D) Nucleus
    E) Mitochondria

21. The process by which phages transmit genetic material from one host to another is known as…
    A) Transmission
    B) Translocation
    C) Transformation
    D) Transmigration
    E) Transduction

22. Genome annotation is the process of ______.
    A) Sequencing an organism’s genome
    B) Identifying genes and start codons, and assigning functions to genes
    C) Assembling an organism’s genome
    D) Producing proteins from genes
    E) Editing an organism’s genome

23. A phage that is *temperate* is one that…
  A) Integrates its DNA in the host's DNA
  B) Reproduces inside its host and lyses its host
  C) Is able to attach to its host
  D) Is unable to attach to its host
  E) Is unable to escape from its host

24. Using the genetic code given below, what is the sequence of the mRNA molecule that codes for the following polypeptide: N-Met-Arg-Ser-Leu-Tyr-C.

	Second Position								
First Position	U		C		A		G		Third Position
U	UUU	Phe	UCU	Ser	UAU	Tyr	UGU	Cys	U
	UUC		UCC		UAC		UGC		C
	UUA	Leu	UCA		UAA	Stop	UGA	Stop	A
	UUG		UCG		UAG	Stop	UGG	Trp	G
C	CUU	Leu	CCU	Pro	CAU	His	CGU	Arg	U
	CUC		CCC		CAC		CGC		C
	CUA		CCA		CAA	Gin	CGA		A
	CUG		CCG		CAG		CGG		G
A	AUU	Ile	ACU	Thr	AAU	Asn	AGU	Ser	U
	AUC		ACC		AAC		AGC		C
	AUA		ACA		AAA	Lys	AGA	Arg	A
	AUG	Met (start)	ACG		AAG		AGG		G
G	GUU	Val	GCU	Ala	GAU	Asp	GGU	Gly	U
	GUC		GCC		GAC		GGC		C
	GUA		GCA		GAA	Glu	GGA		A
	GUG		GCG		GAG		GGG		G

  A) 5'-AUG CGA UCA CUC UGU UGA-3'
  B) 5'-AUG AGA AGC UUG UGU UGA-3'
  C) 5'-AUG AGG UCU UUA GAC UAA-3'
  D) 5'-AUG CGC UCG CUA UAU UAA-3'
  E) 5'-AUG CGG UCC CUU GGU UAG-3'

25. A mutation that results in the change of a single amino acid in a sequence is a ______ mutation.
  A) Insertion
  B) Frameshift
  C) Silent
  D) Missense
  E) Nonsense
